# Attitudes towards transfers of human tissue samples across borders: An international survey of researchers and policy makers in five countries

**DOI:** 10.1186/1472-6939-11-16

**Published:** 2010-09-16

**Authors:** Xinqing Zhang, Kenji Matsui, Benjamin Krohmal, Alaa Abou Zeid, Vasantha Muthuswamy, Young Mo Koo, Yoshikuni Kita, Reidar K Lie

**Affiliations:** 1 Center for Bioethics, Peking Union Medical College, China; 2 Center for Clinical Bioethics, University of Toyama, Japan; 3 Department of Health Science, Shiga University of Medical Science, Japan; 4 Faculty of Medicine, Cairo University, Egypt; 5 Indian Council of Medical Research, India; 6 Department of Medical Humanities and Social Sciences, University of Ulsan College of Medicine, South Korea; 7 Department of Bioethics, Clinical Center, National Institutes of Health, USA; 8 Department of Philosophy, University of Bergen, Norway

## Abstract

**Background:**

Sharing of tissue samples for research and disease surveillance purposes has become increasingly important. While it is clear that this is an area of intense, international controversy, there is an absence of data about what researchers themselves and those involved in the transfer of samples think about these issues, particularly in developing countries.

**Methods:**

A survey was carried out in a number of Asian countries and in Egypt to explore what researchers and others involved in research, storage and transfer of human tissue samples thought about some of the issues related to sharing of such samples.

**Results:**

The results demonstrated broad agreement with the positions taken by developing countries in the current debate, favoring quite severe restrictions on the use of samples by developed countries.

**Conclusions:**

It is recommended that an international agreement is developed on what conditions should be attached to any sharing of human tissue samples across borders.

## Background

Sharing of tissue samples for research and disease surveillance purposes has become increasingly important. The Global Influenza Surveillance Network coordinated by the World Health Organization (WHO) is one such example. In 2007, however, after Indonesia refused to share its H5N1 samples without a legally binding agreement concerning benefit arrangements and appropriate attention to Intellectual Property (IP) rights (patent) issues within the network, WHO initiated a discussion regarding a Pandemic Influenza Preparedness Framework (PIP Framework) to address these concerns[1]. No agreement was reached regarding key issues during deliberations at the World Health Assembly in 2009. The main points of contention are whether a Standard Material Transfer Agreement (SMTA) for sharing of samples within this network should include specific benefit arrangements as conditions of transfer of samples and whether recipients of samples should be free to pursue IP rights to any products developed using the samples obtained through the network. The developed country position is basically that SMTAs should not cover these two issues, whereas the developing country position is that it should.

The Convention on Biological Diversity, which came into force in 1993, contains a section on right of access to genetic resources and the benefits from their use (Article 15). The convention establishes a sovereign right of nations to the genetic resources within their territories and fair and equitable access to benefits arising out of research and commercial use using such resources. Developing countries have referred to this Convention in support of their demand for legally binding agreements regarding transfer of samples, but developed countries have maintained that the Convention is not applicable to the case of influenza viruses. The case is complicated because it is recognized that the Convention on Biological Diversity does not apply to human genetic resources, and the status of flu viruses contained in human tissue is unclear. Currently there is an attempt to develop an International Regime on Access and Benefit Sharing with a draft text expected from a working group sometime in 2010. The issue of benefit sharing in the context of the Convention on Biological Diversity has received quite a bit of attention also in the bioethics literature[2], and there are a few examples of successful negotiations with sponsors for specific benefits from the commercial development of genetic resources[3]. It is unclear, however, whether such examples contain useful lessons for negotiations of access and benefits within more basic research networks. The fact that it has taken a considerable period of time to even develop a draft for an International Regime on Access and Benefit Sharing shows that there is considerable disagreements among the parties.

Sharing of tissue samples among research groups also raises the issue of deciding what research to do on sample collections and who should be authors on papers from such research. Since only a finite number of research projects can be carried out on any given collection of samples, there has to be an agreed on policy with regard to how one should decide what research to approve. Although a number of tissue banks have adopted decision making procedures, there is little guidance and much uncertainty about what substantive criteria should be used to make such decisions[4-6]. This has also been an area of controversy in the case of the PIP Framework.

While it is clear that this is an area of intense, international controversy, there is an absence of data about what researchers themselves and those involved in the transfer of samples think about these issues, particularly in developing countries. In order to begin to explore these issues we carried out a survey (Additional file 1) in a number of Asian countries and in Egypt. The aim of the survey was to identify what policy makers, researchers and members of research ethics review committees thought about key issues related to access to stored tissue samples.

## Methods

### Survey countries and populations

The target populations were enrolled from sites in China, Egypt, India, Japan, and South Korea. The countries were chosen based on an existing network of researchers in these countries. The potential participants were selected from the following four groups, 1) researchers who have been or are conducting research on human biological samples, 2) collectors who have been or are collecting human biological samples, 3) ethics committee members who are currently sitting as a research ethics review board member, and 4) policy-makers who have been involved in setting an institution's policy with regard to research on stored tissue samples. Local PIs in each country determined the way of enrolling research participants, and therefore the participants other than the Japanese participants who were enrolled through cluster randomization, were a sample of convenience.

### Questionnaire

For details regarding the questionnaire development, see the publication of results from the first part of the survey on issues related to informed consent[7]. The questionnaire was translated and administered in the local language. The part related to international transfer of samples contained the following four survey domains which assess participant attitudes towards:

1. Decisions regarding location of samples

2. Decision making procedures for choice of research on samples

3. Issues related to authorship of publications

4. Issues related to intellectual property rights

Most of questions were in the form of a binary choice or a five-point Likert scale ranging from 1 (strongly disagree) to 5 (strongly agree). The survey was conducted between 2005 and 2008. The term "local scientist" refers to scientists who live and oversee research and collection in the country where samples are taken. The term "foreign collaborating scientist" refers to scientists from other countries.

### Human subjects approval

The ethics approval at the US National Institutes of Health was formally exempted by Office of Human Subjects Research (No. 3074). Each collaborating local PI obtained an ethics approval from a research ethics committee of her own institution.

## Results

The total number of valid responses obtained was 154 in China, 186 in Egypt, 127 in India, 864 in Japan, and 105 in Korea. The response rate for Japan in which the questionnaires were sent out to the potential participants of randomly selected institutions was approximately 33%. For the other three countries where the potential participants were of a sample of convenience, no detailed data about response rate were available.

### Demographic characteristics

Compared to the other three countries, the respondents in India and Japan were relatively older. Most of the respondents except the Chinese had doctoral degrees. Among all of the respondents, there were 341 EC members, 23.7% of the sample. About a quarter to a half of the respondents in each country reported that they were currently involved in policy making processes concerning research. Other than the Japanese respondents, a majority reported that they were conducting research on stored human biological samples (from 57.5% in India to 89.6% in China) and collecting them for future use in research (from 56.7% in India to 67.2% in Egypt), whereas doing so among the Japanese respondents were only 35.1% and 29.1%, respectively. For additional details regarding demographics see the companion publication[7].

### Involvement in the use of Material Transfer Agreements

Most of our respondents had not been involved at all in the use of a Material Transfer Agreement (MTA) for the transfer of biological samples (81.9% of respondents, varying from lows of 47.4% in China and 61.4% in India to a high of 93.3% in Korea). In China and India most of those who had been involved in MTAs had been involved in the development of the MTA itself or the transfer of samples (27.3% for China and 15% for India, and 27.3% for China and 26.8% for India respectively).

### Intellectual property, royalties and benefit arrangements (Table 1)

**Table 1:** Numbers and percentages strongly agreeing or agreeing to the statements regarding who should receive benefits from research on stored tissue samples

MTAs should require that, foreign collaborating scientists share royalties from discoveries, patents and intellectual property that arises from research on the samples. ...	China n = 154	Egypt n = 186	India n = 127	Japan n = 864	Korea n = 105
With local scientists	107 69.5%	162 87.1%	102 80.3%	430 49.8%	75 71.4%

With the population or country from which the samples were taken	95 61.7%	129 69.4%	100 78.7%	302 35%	55 52.4%

MTAs should require that the population or country from which the samples were taken is given access to material products such as pharmaceuticals, that arise from research on the samples	118 76.6%	166 89.2%	102 80.3%	407 47.2%	73 69.5%

Local scientists are under pressure to accept unfavorable conditions for the transfer of their sample collections to foreign collaborating scientist with access to more resources	13 8.4%	78 42%	41 32.2%	187 21.6%	66 62.8%

The respondents were asked about how intellectual property rights related to research on the samples should be handled. There was general agreement that royalties should be shared with the local scientists, ranging from a high of 87.1% in Egypt, to 49.8% in Japan. Smaller percentages in all countries agreed that royalties should be shared with the local population, ranging from 78.7% in India to 35% in Japan. There was also a general agreement that the population from which the samples were taken should given access to products, such as a vaccine or new drug, that arise from research on the samples, ranging from 47.2% in Japan to a high of 89.2% in Egypt.

### Location of collected samples (Table 2)

**Table 2:** Numbers and percentages strongly agreeing or agreeing to the statements regarding where stored tissue samples should be located

	China n = 154	Egypt n = 186	India n = 127	Japan n = 864	Korea n = 105
Samples should always be kept in the country where they were collected	60 38.9%	150 80.6%	61 40.1%	170 19.7%	32 30.4%

Samples should only be transferred when research facilities are unavailable in the country of origin	91 59.1%	142 76.3%	99 78%	192 22.2%	45 42.9%

If samples are removed, a portion must be left behind so that local scientists can use them for their own research, unless special government permission is granted	122 79.2%	149 80.1%	105 82.7%	369 42.7%	60 57.2%

Opinions overall were almost evenly divided about the question of where collected samples should be stored, with 32% agreeing with the statement that samples should always be kept in the country where they were collected and 40% disagreeing with the statement. There were, however, marked differences between the countries, with only 10.2% in Egypt disagreeing with this statement compared with 47.2% in Japan, and 48.8% in Korea.

We asked two questions about specific conditions for when it might be reasonable to move samples out of a country. Sometimes appropriate facilities are not available in the country of origin to do important research. The question, however, was phrased in such a way that we asked the respondents what they felt about this being the only condition for transfer of samples out of the country. In all countries, except in Korea and Japan, a high number of respondents agreed that this should be the only condition.

It has been proposed that a portion of the sample could be left behind when it is necessary to do an analysis outside the country. We asked respondents about their attitude towards this policy proposal. Again, the acceptability of such proposal was higher in the four developing countries, compared with the respondents in Japan and Korea.

### Decisions about what research to do on stored samples (Table 3)

**Table 3:** Numbers and percentages strongly agreeing or agreeing to the statements regarding how decisions for future research should be made

	China n = 154	Egypt n = 186	India n = 127	Japan n = 864	Korea n = 105
MTAs should require that foreign collaborating scientists consult local scientists before any new use of samples	138 89.6%	170 91.4%	118 92.9%	1011 67.7%	82 78.1%

MTAs should require local scientists to have some decision making power over the future use of samples	132 85.7%	169 90.9%	108 85%	484 56.1%	76 72.3%

MTAs should require that decisions regarding future use of samples should be made jointly by a committee composed of representatives of both local and foreign scientists	110 71.4%	162 87.1%	106 83.5%	461 53.4%	72 68.5%

MTAs should require that local scientists have veto power over any future use of samples by foreign collaborating scientists	98 63.7%	156 83.8%	88 69.2%	410 47.5%	73 69.5%

MTAs should require that a local scientist is involved in the protocol development team for any future research on the samples	130 84.4%	165 88.7%	103 81.1%	457 52.9%	70 66.2%

We next asked about opinions regarding decision making authority over the stored samples. When samples are stored for future research, decisions have to be made about what research should be done on such samples in the future. We asked respondents to consider various alternatives, giving different levels of control over the samples to local scientists.

The weakest involvement of local scientists would require a consultation with them before any research is done. There was general agreement among all respondents in all countries that this should be required, varying from a high of 92.9% in India to a low of 67.7% in Japan.

Next we asked whether local scientists should have some decision making power over the use of the samples. A smaller percentage of respondents in all countries agreed with this statement, varying from 90.9% in Egypt to 56.1% in Japan.

We asked specifically whether there should be a decision making committee comprised of representatives from the sending and the recipient countries. A high percentage from all countries except Japan agreed with this statement.

The strongest control over the use of the sample by local scientists would be if they have a veto power over any use of such samples. Here there was much more divergence of opinions, where only Egypt still had a high percentage of respondents agreeing with this position (83.8%), whereas in Korea 69.5%, in India 69.2%, in China 63.7% and in Japan 47.5% agreed with this proposal.

Finally, we asked whether local scientists should always be included on any future protocol team. Here again, there was wide agreement in all countries, but with lower percentages agreeing in Japan (52.9%), and Korea (66.2%) than in the other countries, with the highest again being Egypt (87.1%).

### Issues of authorship (Table 4)

We asked how collaborating scientists should handle the issue of authorship. Specifically, we asked how Material Transfer Agreements should handle this issue. Table 4 gives the results regarding these questions. We obtained a range of answers from the respondents in different countries. Regarding the question whether local scientists should be authors on all papers arising from research on the samples, the agreement ranged from a high of 78.5% in Egypt to a low of 22% in Japan, with around half agreeing in China, India and Korea. There was a higher degree of agreement in all countries, except Korea, on whether local scientists should be the author on the first paper arising from the research, ranging from a high of 60.4% in China to a low of 27.6% in Japan. There was general agreement in all countries that scientists should only be authors if they provide enough intellectual input to the publication, ranging from a high of 60.4% in China to a low of 43% in Japan.

**Table 4:** MTAs should require that, in exchange for providing the samples, local scientists are credited for authorship

	China n = 154	Egypt n = 186	India n = 127	Japan n = 864	Korea n = 105
On all publications arising from research on the samples	65 42.2%	146 78.5%	74 58.3%	190 21%	51 48.6%

On the first publication arising from research on the samples	93 60.4%	101 54.3%	52 40.9%	239 27.6%	43 41%

Only if local scientists provide sufficient intellectual input into the publication	93 60.4%	90 48.4%	76 59.8%	371 43%	59 56.2%

MTAs should require that local scientists be given the opportunity to provide sufficient intellectual input to be credited for authorship on publications arising from research on the samples	113 73.3%	169 90.9%	110 86.6%	445 51.5%	63 60%

Numbers and percentages strongly agreeing or agreeing to the statements regarding how decisions for authorship on papers arising from the research should be decided.

Finally, we asked the question whether the respondents thought that MTAs should require that local scientists be given the opportunity to provide sufficient intellectual input so that it would be justified to credit them for authorship. There was overwhelming agreement regarding such a requirement, ranging from 90.9% in Egypt to 51.5% in Japan.

There are some differences in the answers to these questions with regard to experience with MTAs. For example, among those who have been involved in the use of MTAs as a receiver of samples, only 33.3% agree that local scientists should be authors on all papers arising from the samples, whereas this agreement is at 49.3% among those who have been involved in the transfer of samples. Among those who have been involved in the development of MTAs there is an intermediate agreement at 36.2%.

### Legally binding regulations and role of local scientists

We asked questions about respondent attitudes towards binding regulations regarding rights of local scientists. Again, there was general agreement that binding regulations should be in place to ensure that the rights of local scientists are protected, ranging from 50.5% in Japan, 76.6% in China, 81% in Korea, 89% in India, and 95.1% in Egypt. We asked who should keep these regulations to protect local scientists. Here there was a wide divergence between countries. Around half of respondents in all countries, except India where only 20% agreed, thought that the World Health Organization should do so. Most countries, except Japan, thought that either the local government or the local institution should do so. In Egypt, most favored the local institution, rather than the local government.

Finally, we asked the question about their perception regarding pressure to accept unfavorable conditions when negotiating MTAs. For all countries except Korea, few respondents agreed that local scientists are under pressure to accept unfavorable conditions for the transfer of samples, ranging from a low of 8.4% in China to high of 62.8% in Korea, with Japan, Egypt and India ranging from 21.6% to 42%.

## Discussion

The choice of countries for this survey was not motivated by a desire to explain the controversy over access to a pandemic flu vaccine. Nevertheless it is interesting to note how the responses in our survey map the positions taken by representative countries in the current controversy over access to pandemic flu vaccines. Our study demonstrates broad agreement for the developing country position in the current controversy over SMTAs within the PIP framework. The respondents would want IP rights to be shared with researchers or the source country, and favor access to products resulting from research on the samples. This is, not surprisingly, most evident among developing country researchers, where as many as 80% are in favor of these positions. But the support is also surprisingly high in Japan, a representative of a developed country, where 35% think that royalties should be shared with the population of the source country. 47% of our Japanese respondents believe that MTAs should require that the source country should be given access to material products such as pharmaceuticals. If our data are representative of the positions taken by researchers and ethics review committee members in these countries, it indicates that there is no broad agreement for the position taken by developed countries in the ongoing debate within WHO.

Developed countries, primarily represented by the EU and the US, have consistently taken the position during the debate within WHO that SMTAs should not contain legally binding benefit arrangements nor restrictions on IP rights. At most, there can be reference to guidelines that suggest appropriate benefits to source countries[8]. Even the so-called "middle position" suggested by the WHO secretariat has consistently sided with developed countries in this regard. The rationale for this position is that strong IP rights are necessary to motivate vaccine R&D, which ultimately will benefit developing countries. Developing country representatives have consistently complained that their views have not been adequately incorporated into the drafts of the SMTAs, and have maintained that they should contain legally binding benefit arrangements and should not allow recipients of samples to pursue IP rights on products developed using the samples. The rationale for this position is that the recent experience with the H1N1 pandemic has demonstrated that pandemic flu vaccines are accessible to developing countries only after supply to developed countries have been secured[9]. Our data demonstrate considerable sympathy for the developing country position among our respondents.

Although IP issues and access to material benefits have been the focus of discussion within the PIP framework during World Health Assembly (WHA) meeting during the past couple of years, developing countries have also voiced other concerns in the debate, although these have not been discussed as extensively. For example, according to the WHA resolution 60.28 in 2007, SMTAs should be based on the principles of "increased involvement, participation, and recognition of contribution of scientists from originating country in research related to viruses and specimens and attribution of the work and increased co-authorship of scientists from originating countries in scientific publications"[1]. Following up on this resolution, a proposal from Thailand specified that before publication of results of research on donated samples, the source country should be consulted and not object to the publication[10]. Similarly, a proposal from several African countries also required prior informed consent from the donating country, as well as a requirement of involvement of source country researchers in the execution of the research and publication of results[11]. None of these proposals have been followed up by the WHO secretariat.

Our data again support the positions taken by these countries (table 3). For example, a substantial number of our respondents favor an essential veto power of the source country over any future use, ranging from 47.5% in Japan to 83.8% in Egypt. This very restrictive position is in line with the suggestion by developing country that the source country will have to approve any research publication arising out of the use of the samples. There is even wider agreement for more moderate positions, such as involving local scientists in protocol development, or jointly deciding which research should be done on the samples.

Interestingly, our respondents also favor legally binding regulations for the transfer of samples to protect the rights of local scientists. Representatives from developing countries have insisted throughout the discussion on the PIP framework that SMTAs should include legally binding provisions for benefit arrangements as well as restrictions on IP rights. In contrast, developed countries and to a certain extent the WHO secretariat have insisted that benefit arrangements and IP rights should only be referred to in guidelines. This basic disagreement has to a certain extent paralyzed the negotiations, where each side insists on maintaining their positions. Our data demonstrate widespread sympathy for the developing country position among our respondents.

The debate over SMTAs in the context of Pandemic Influenza Preparedness and the results from our survey raise the question of how one should move the agenda forward and deal with the impasse reached in the negotiations. Two points seem especially important.

One the one hand, some of the suggestions from developing countries and our respondents for specific provisions in an SMTA seem difficult to defend. For example, it does not seem justifiable to demand that source countries or local scientists should have veto rights over any publications resulting from use of stored tissue samples. At least sometimes, this could be analogous to a sponsor, such as a pharmaceutical company, requiring collaborating scientists to sign agreements where they can only publish after consent of the sponsor, leading to a justifiable criticism that this could lead the sponsor to suppress results unfavorable to the sponsor. Similarly, what restrictions one should place on IP rights seem to a large extent to be a matter of what mechanism is best suited to stimulate innovations of products that will have major health benefits. Although there will be disagreements about specifics, it should be possible to have a discussion of the merits of various proposals.

On the other hand, it does not seem prudent for developed countries to insist that substantive provisions for benefits should be kept out of SMTAs. Developing countries have continued to insist on their inclusions but their position has been rejected by developed countries and the secretariat. The WHO secretariat should probably recognize the widespread support of the position taken by developing countries, which is also evident from the data in our survey. Previous surveys in Europe have documented considerable worries about commercialization of research on stored samples, both among those involved in biobanks[4] as well as among the general population[12]. Rather than therefore to reject the inclusion of binding benefit arrangements in the SMTA, the starting point should be their inclusion. Once the principle has been accepted, one can start on working out the details of the provisions.

This study has several limitations. First, we assessed the choices of survey respondents, most of whom were a sample of convenience. As a result, our findings may be biased toward particular groups of samples and may not be generalizable to other populations or other countries. Second, the small sample of developed and developing countries. surveyed may not be generalizable to developed and developing countries as a whole, respectively. Finally, since we did not probe for reasons for answers from the respondents, it is unclear whether the respondents had motivations besides those mentioned in the discussion for answering as they did.

## Conclusions

In conclusion, this study demonstrates that there here is substantial agreement amongst all respondents to favor some rights for local scientists and to share in the benefits of research. As seen in the Indonesian case and elsewhere, answers for how to arrive at an agreement for elements of MTAs are urgently needed. Our data also show that there is wide variation in attitudes on this subject between countries and professional groups. This points to a need to explore the sources of disagreement and to develop a coherent framework for understanding benefit sharing and elements of MTAs.

When moving forward it may also be important not to focus exclusively on the most difficult parts, namely guaranteed access to product developed using provided tissue samples or issues of IP rights. As the discussion within WHO and the responses to our survey show, there are other contentious issues as well: who decides and based on what criteria does one decide how the samples should be used, and who should receive credits on publications arising out of the research. Specific proposals have been put forward by a variety of developing countries, but have not been taken up in the discussion. Interestingly, these are also issues that are unresolved for tissue banks established in developed countries. A recent report commissioned by the UK Medical Research Council and the Wellcome Trust recommended that a standardized access policy to sample collections be developed[5]. Recently, the UK National Cancer Research Institute has developed a template for agreements regarding access policies for tissue banks, after an extensive consultation process[13]. The template covers issues such as conditions for dissemination of results of research. Developing a similar framework within the international context such as PIP could build on these efforts, and they demonstrate that some agreement is possible.

## Competing interests

The authors declare that they have no competing interests.

## Authors' contributions

All authors were involved in the design of the questionnaire. The authors in the countries in which the questionnaire was administered were responsible for translation of the questionnaire to the local language, administration of the questionnaire, and data entry. All authors were involved in the writing of the paper and the analysis of the data. All authors have read and approved the final manuscript.

## Pre-publication history

The pre-publication history for this paper can be accessed here:

http://www.biomedcentral.com/1472-6939/11/16/prepub

## Supplementary Material

Additional file 1 **Surveyinstrument**. This file contains the survey instrument. Click here for file
