# Supplementary material for: Attitudes towards transfers of human tissue samples across borders: An international survey of researchers and policy makers in five countries
Source: BMC Med Ethics. 2010 Sep 16;11:16. doi: 10.1186/1472-6939-11-16 (PMC2949780; doi:10.1186/1472-6939-11-16)
Supplement: Additional file 1 — Surveyinstrument. This file contains the survey instrument. [file 1472-6939-11-16-S1.DOC]

**International Survey of Scientist and Policy Makers’ Attitudes Toward Research on Stored**

**Human Biological Materials**

**___________________________**

**Respondent Identification Code**

**____/______/________**

**Day Month Year**

**______________________**

**Country**

(Mark an  for the appropriate answer.)

**A. Personal Background Information**

1. Gender:

Male ……………………… 1

Female ……………………… 2

2. Age in years _______

3. Current area of work (**check all that apply**):

Law ……………………… 1

Clinical medicine ……………………… 2

Scientific research ……………………… 3

Administration ……………………… 4

Health policy ……………………… 5

Social science ……………………… 6

Philosophy ……………………… 7

Other (specify below) ……………………… 8

________________________________________

4. How many years have you worked in your current area of work?

_______

5. Current employer (**check all that apply**):

Private research institution…………………… 1

Public research institution….………………… 2

Hospital or clinic ……………………… 3

Local government ……………………… 4

National government ……………………… 5

International organization….………………… 6

University ……………………… 7

Ethics committee ……………………… 8

6. Degrees (**check all that apply**):

Bachelors ……………………… 1

Masters ……………………… 2

PhD ……………………… 3

MD ……………………… 4

JD ……………………… 5

Other (specify below) ……………………… 6

________________

7. As part of your current job(s), are you involved in making policy or recommendations regarding the use of stored human biological samples?

yes ……………………… 1

no ……………………… 2

8. As part of your current job(s), do you conduct research on stored human biological samples?

yes ……………………… 1

no ……………………… 2

9. As part of your current job(s), do you collect human biological samples for use in future research?

yes ……………………… 1

no ……………………… 2

10. Have you ever donated biological samples for research purposes?

yes ………………………. 1

no……………………………………………… 2

11. Have you worked in collaborative partnerships with people in countries other than the country where you currently work?

yes…………………………………………….. 1

no…………………………………………… 2 →skip to question 13

12. Have you collaborated with people in:

only developing countries…………………….  1

only developed countries……………………... 2

both developing and developed countries……. 3

13. Have you ever worked in a country other than the country in which you currently work?

yes……………………………………………... 1

no ………………………. 2

14. Have you worked in:

only developing countries…………………….  1

only developed countries……………………... 2

both developing and developed countries……. 3

**B. Consent Policy**

**Consent Options**

**Before human biological samples may be collected and stored for research purposes, donors must provide consent for their samples to be stored and analyzed for the research project for which they are being recruited. In some cases, donors may also be given the option to consent in advance to the use of their samples in future research. There is a debate about which options for consent to future research, if any, should be offered to donors at the time of sample collection.**

**Some argue that donors should not be allowed to consent to any future research on their samples. These people argue that researchers must re-contact donors to obtain consent for any future research to be done on the donor’s samples.**

**Others argue that donors should sometimes be allowed to consent to future research on their samples.**

**Assume for all of section B that all research on human samples will be approved by an ethics committee. Indicate which of the following you agree with most by marking an**  **for the appropriate answer.**

15. Consent forms for the storage of samples intended for future research should:

Never provide donors with the option to consent

to future research………………………………1

Sometimes provide donors with the options to consent

to future research ……………………..………..2

Do not know/no opinion………………………………….. 3

**When donors are given the option to consent to future research on their samples, there is disagreement about whether consent forms should present donors with many consent options, allowing them to specify types and conditions of future research for which their samples may be used, or whether consent forms should only present donors with the choice to consent or not to consent to future research. Some argue that having many options is better for donors, while others argue that a simple yes/no choice is less confusing for donors and more convenient for researchers. Assume that it is the policy of your institution’s research ethics committee to allow consent for future research, and that you have been asked to provide more specific policies for what should be on the informed consent form. Indicate which of the following you agree with most by marking an**  **for the appropriate answer.**

16. Consent forms that give donors the option to consent to future research should:

Include multiple options regarding the types and conditions

of future research for which the samples may be used……...1

Include two options, to either consent or not consent to

all future research on the samples……………...……………2

Do not know/no opinion…………………………………….3

**Imagine you are writing a consent form for donors to consent to the use of their samples in future research, providing them with more specific options. Indicate which of the following options for donors you would include in the consent form.**

|  | Include | Don’t Include |
| --- | --- | --- |
|  | ↓ | ↓ |

The option to provide consent to future research . . .:

17. For any ethics committee approved research……………..1………………2

18. Only after re-contact by researchers…………………..…..1………………2

19. If the samples do not retain personal identifiers. …………1………………2

20. If donors will be notified of research findings

that may be relevant to the donor’s health. ……………….......1……………….2

21. If the donor can later withdraw his or her

samples from use in research. ………………………………...1……………….2

22. If it is sponsored by not-for-profit organizations. ……......1……………….2

23. If the research pertains to a particular disease..…………...1……………….2

24. If the samples will remain in a specified

country or region…………….……………………………..….1……………….2

25. If the research will be

performed by specified researchers or institutions ...………….1……………….2

26. Please specify any other options that you would include in the consent form:

________________________________________________________________________________________________________________________________________________________________________________________________________________________________________________________________________________________________

**Reasons**

**This section asks for your opinion on reasons for and against providing the option to consent to the use of samples in future research. Indicate the degree to which you agree or disagree with the following statements by checking one of the options, 1 through 5. 5 indicates strong agreement, 1 indicates strong disagreement, and 3 is neutral.**

|  | Strongly disagree |  | Neutral |  | Strongly agree |
| --- | --- | --- | --- | --- | --- |

|  | 1 | 2 | 3 | 4 | 5 |
| --- | --- | --- | --- | --- | --- |
|  | ↓ | ↓ | ↓ | ↓ | ↓ |

***Requiring researchers to re-contact***

***donors before using the donors’ samples***

***in research would:***

27. Place an unacceptable burden on researchers………1….2…..3.…4…..5

28. Be prohibitively costly for researchers…………..…1….2…..3.…4…..5

29. Be an inconvenience to donors……………..………1….2…..3.…4…..5

***Donors’ consent to future***

***research on their samples:***

30. Does not provide donors with enough

information to give meaningful consent..……….………1….2…..3.…4…..5

31. Does not show enough respect for donors. ..…….…1….2…..3.…4…..5

32. Does not provide enough protection for donors..…..1….2…..3.…4…..5

33. Does not provide donors with enough control

over the future use of their samples. ..……………..……1….2…..3.…4…..5

**Some people think that donors should NOT be able to give advance consent to future research on their samples, except** under certain conditions. Indicate the extent to which you agree or disagree with the following claims about this issue.

|  | Strongly disagree |  | Neutral |  | Strongly agree |
| --- | --- | --- | --- | --- | --- |

|  | 1 | 2 | 3 | 4 | 5 |
| --- | --- | --- | --- | --- | --- |
|  | ↓ | ↓ | ↓ | ↓ | ↓ |

***Donors should only be able to provide***

***consent to future research***

***on their samples:***

34. If their samples will be anonymous and

unlinked to identifying information……………………..1….2…..3.…4…..5

35. If donors will have the opportunity to withdraw

consent later on. ..……………………………..…..……1….2…..3.…4…..5

36. If the donor will be notified if any

information relevant to the donor’s health is discovered…………………….……………..………...1….2…..3.…4…..5

37. There should be no limitation on the

advance consent that donors may provide,

so long as future research on their samples is

approved by an ethics committee..……………..………1….2…..3.…4…..5

**C. Rights of Local and Foreign Collaborating Scientists**

**Scientists in different countries often collaborate on research involving the collection of human samples. Therefore, samples are often collected in one country, and sent to be stored in another country. Transfer of samples is usually governed by a Material Transfer Agreement (MTA) which specifies collaborators’ rights and responsibilities with respect to the collection of samples. The questions and statements in the following section will ask for your opinion and the extent to which you agree or disagree with statements about this issue.**

**We will use the term “local scientist” to refer to scientists who live and oversee research and collection in the country where samples are taken. We use the term “foreign collaborating scientist” to refer to scientists from other countries.**

**Indicate the degree to which you agree or disagree with the following statements about this issue by checking one of the options, 1 through 5. 5 indicates strong agreement, 1 indicates strong disagreement, and 3 is neutral.**

|  | Strongly disagree |  | Neutral |  | Strongly agree |
| --- | --- | --- | --- | --- | --- |

|  | 1 | 2 | 3 | 4 | 5 |
| --- | --- | --- | --- | --- | --- |
|  | ↓ | ↓ | ↓ | ↓ | ↓ |

38. Samples should always be kept in the country

where they were collected. ……..……………..………...1….2…..3.…4…..5

39. Samples should only be transferred when research

facilities are unavailable in their country of origin……...1….2…..3.…4…..5

40. If samples are removed from their country of origin,

a portion must be left behind so that local scientists can

use them for their own research, unless special

governmental permission is obtained………....………...1….2…..3.…4…..5

41. MTAs should require that foreign collaborating

scientists consult local scientists before any new use

of the samples in research. ……..……………………….1….2…..3.…4…..5

42. MTAs should require that local scientists have

some decision-making power regarding the future use

of samples. ……..…………………………..………......1….2…..3.…4…..5

43. MTAs should require that decisions regarding

future use of samples be made jointly by a committee

composed of representatives of both the local

scientists and the foreign collaborating scientists. .…...1….2…..3.…4…..5

44. MTAs should require that local scientists have

veto power over any future use of samples by the

foreign collaborating scientists. ……..………………...1….2…..3.…4…..5

45. MTAs should require that a local scientist is

involved in the protocol development team for any

future research on the samples. ……………..………...1….2…..3.…4…..5

***MTAs should require that, in exchange for providing***

***the samples, local scientists are credited for authorship:***

46. On all publications arising from

research on the samples. ……..……………..………...1….2…..3.…4…..5

47. On the first publication arising from

research on the samples. ……..……………..………...1….2…..3.…4…..5

48. Only if local scientists provide sufficient

intellectual input into the publication. ……..………...1….2…..3.…4…..5

________________________________________________________________________

49. MTAs should require that local scientists be

given the opportunity to provide sufficient

intellectual input to be credited for authorship

on publications arising from research on the

samples. ……..……………………………..………...1….2…..3.…4…..5

***MTAs should require that foreign***

***collaborating scientists share royalties from***

***discoveries, patents, and intellectual property***

***that arises from research on the samples:***

50. With local scientists..………….………..………..1….2…..3.…4…..5

51. With the population or country

from which the samples were taken……….................1….2…..3.…4…..5

________________________________________________________________________

52. MTAs should require that the population

or country from which the samples were taken

is given access to material products, such as

pharmaceuticals, that arise from research on the

samples……………….……..……………..………...1….2…..3.…4…..5

53. Local scientists are under pressure to accept

unfavorable conditions for the transfer of their

sample collections to foreign collaborating scientists

with access to more resources……..…..…..………...1….2…..3.…4…..5

54. Binding regulations should be in place to ensure

that certain protections for local scientists are

included in MTAs.……..………………....………...1….2…..3.…4…..5

55. Who do you think should keep regulations in place to protect the interests of local scientists who transfer biological sample collections to others? **Check all that apply.**

There should be no such regulations;

scientists should be able to make MTAs as they see fit…….… 1

The World Health Organization……………………………..… 2

The national government of the local scientists………………... 3

The national government of the receiving scientists ………….. 4

The research or clinical institutions where the local

scientists are employed………………………………………… 5

Other (specify below)…………………..……………………… 6

____________________________________________________________________

____________________________________________________________________

56. Have you ever been involved in the use of an MTA for the transfer of human biological samples? (**check all that apply**)

No ……………………… 1

Yes, in the development of the MTA………… 2

Yes, as one of those receiving samples………. 3

Yes, as one of those transferring samples…….. 4

57. Please write any comments you might have about your experience with MTAs:

____________________________________________________________________

____________________________________________________________________

____________________________________________________________________

____________________________________________________________________
